# Optimization of adipose tissue-derived mesenchymal stem cells by rapamycin in a murine model of acute graft-versus-host disease

**DOI:** 10.1186/s13287-015-0197-8

**Published:** 2015-10-23

**Authors:** Kyoung-Woon Kim, Su-Jin Moon, Min-Jung Park, Bo-Mi Kim, Eun-Kyung Kim, Sung-Hee Lee, Eun-Jung Lee, Byung-Ha Chung, Chul-Woo Yang, Mi-La Cho

**Affiliations:** Convergent Research Consortium for Immunologic disease, Transplant Research Center, The Catholic University of Korea, Seoul, South Korea; Division of Rheumatology, Department of Internal Medicine, College of Medicine, The Catholic University of Korea, Seoul, South Korea; The Rheumatism Research Center, The Catholic University of Korea, Seoul, South Korea; Division of Nephrology, Department of Internal Medicine, College of Medicine, The Catholic University of Korea, Seoul, South Korea; Department of Internal Medicine, Seoul St. Mary’s Hospital, 505 Banpo-Dong, Seocho-Ku, 137-040, Seoul, Korea

## Abstract

**Introduction:**

Mesenchymal stem cells (MSCs) can protect bone marrow transplantation (BMT) recipients from the lethal acute graft-versus-host disease (aGVHD) development. However, the mechanisms underlying the anti-inflammatory properties of MSCs in aGVHD remain to be elucidated. The immunoregulatory properties of MSCs are mediated by their production of anti-inflammatory molecules, including IL-10 and TGF-β. On the other hand, MSCs can also produce proinflammatory cytokines during their normal growth, such as IL-1β and IL-6. These opposite actions may limit their therapeutic application in aGVHD. Therefore, optimization of the functional properties of MSCs can increase their benefits.

**Methods:**

The expressions of mRNA and protein were analyzed by real-time PCR and western blotting, respectively. Expression of MSC markers was assessed by flow cytometry. An animal model of aGVHD was established by transplanting C57BL/6 donor bone marrow cells and spleen cells into lethally irradiated BALB/c recipient mice. The recipient mice were divided into the control group and the therapy [adipose tissue-derived human MSCs (Ad-hMSCs) or rapamycin-treated Ad-hMSCs] groups. The survival, body weight and clinical score of aGVHD in transplanted mice were monitored.

**Results:**

Rapamycin pre-treatment of Ad-hMSCs increased mRNA synthesis of IL-10, indoleamine 2,3-dioxygenase, and TGF-β compared with untreated Ad-hMSCs. Rapamycin-treated Ad-hMSCs suppressed clonal expansion of interleukin-17-producing CD4^+^ T (Th17) cells more effectively than untreated cells. mRNA expression of autophagy markers such as ATG5, LC3A and LC3B was significantly increased in the rapamycin-treated Ad-hMSCs compared with untreated Ad-hMSCs. Transmission electron microscopy revealed that Ad-hMSCs exposure to rapamycin resulted in the appearance of autophagic vacuoles. Interestingly, *in vitro* migration efficiency of rapamycin-treated Ad-hMSCs toward the CD4^+^ T cells was increased significantly compared with the untreated cells. And, these effects were associated with autophagy induction capacity of rapamycin. *In vivo*, the inhibiting properties of MSCs on the clinical severities of aGVHD were greater in the mice receiving rapamycin-treated Ad-hMSCs compared with untreated Ad-hMSCs. The beneficial effects of rapamycin treatment in Ad-hMSCs shown *in vivo* were associated with a reduction of Th17 cells and an increase in regulatory T cells.

**Conclusions:**

Rapamycin can optimize the immunomodulatory potential of Ad-hMSCs, suggesting a promising strategy of MSC use in aGVHD treatment.

## Introduction

Allogeneic bone marrow transplantation (BMT) has been used to treat malignant and nonmalignant hematological diseases. Despite improvements in conditioning regimens and graft-versus-host disease (GVHD) prophylaxis, the development of acute graft-versus-host disease (aGVHD) remains a lethal and significant complication in allogeneic BMT recipients, limiting the application and efficacy of allogeneic BMT [1]. Indeed, GVHD develops in approximately 40–60 % of recipients [2]. Although the pathophysiology of aGVHD is not yet understood fully, immunobiological research has shown GVHD to be a complex inflammatory process, arising due to activation and expansion of donor T cells and the production of proinflammatory cytokines and chemokines, which increase the expression of key receptors on host antigen-presenting cells (APCs), thereby enhancing cross-presentation of polypeptide proteins to the donor immune cells [3].

Based on their immunoregulatory properties, which have been established by pre-clinical studies [4, 5] as well as by several clinical studies [6, 7], mesenchymal stem cells (MSCs) are being widely studied as a promising platform for cell-based therapy to prevent or treat aGVHD. MSCs can decrease GVHD when co-transplanted with hematopoietic stem cells [8]. Previous case reports demonstrated that grade IV therapy-resistant aGVHDs involving the gut and liver were rapidly improved after infusion of bone marrow-derived MSCs (BM-MSCs) that were isolated from the haploidentical donor, suggesting the therapeutic potential of third party MSCs [9]. Within the context of an adaptive immune system, BM-MSCs can suppress alloreactive T-cell proliferation via contact-dependent mechanisms and production of soluble factors, such as IL-10 [10]. MSCs are present in various tissues including bone marrow, peripheral blood, umbilical cord blood, and adipose tissues and possess intrinsic immunoregulatory properties that modulate innate and adaptive immune systems [11–14]. When compared with BM-MSCs, adipose tissue-derived human MSCs (Ad-hMSCs) might serve as an alternative with some advantages; adipose tissue can be obtained in a minimally invasive manner, such as lipectomy or liposuction. In addition, previous in vitro studies have established that Ad-hMSCs also have similar or even higher immunoregulatory capacity, when compared with BM-MSCs [15].

Apart from the immunosuppressive activity of MSCs, the cells were shown to secrete various growth factors, such as vascular endothelial growth factor (VEGF) [16] and proinflammatory cytokines, such as IL-6 [17]. Serum level of VEGF in recipients was suggested as a predictor for the emergence of GVHD, suggesting the potential as a principal factor regulating immune reactions associated with acute and/or chronic GVHD [18]. IL-6 has recently been proposed as a key mediator in the pathogenesis of GVHD in an experimental model [19, 20]. Thus, the growth factor and inflammatory cytokines secreted by MSCs might raise doubts about the clinical application of MSCs in human GVHD.

Rapamycin was used as an immunosuppressive agent for the prevention of acute rejection in renal transplantation [21]. Despite wise use of the drug, rapamycin has adverse effects, including a toxic effect on pancreatic β-cells [22], dyslipidemia [23], and pulmonary toxicity [24], thereby limiting the use of the drug in organ transplant recipients. Interestingly, rapamycin acts by binding to the mTOR FK506 binding protein complex leading to inhibition of mTOR protein kinase activity. Once mTORC1 is inactive, autophagy is initiated, in part through phosphorylation of Beclin-1, leading to maturation of the autophagosome from the endoplasmic reticulum [25]. Several recent studies have revealed that autophagy induction in MSCs can protect these cells from undergoing apoptosis, leading to enhanced MSC survival [26]. Several recent studies have suggested that the management of autophagy in MSCs could optimize their clinical applications [27, 28]; however, whether autophagy induction in MSCs influences GVHD prevention is unclear. In this study, we demonstrated that autophagy induction in adipose tissue-derived human MSCs (Ad-hMSCs) by rapamycin treatment enhanced their immunoregulatory properties and reciprocally inhibited the production of proinflammatory cytokines. Interestingly, the migration capability of Ad-hMSCs was significantly enhanced by rapamycin treatment, and these effects were dependent on autophagy induction. In vivo, rapamycin-treated Ad-hMSCs showed more potent inhibition of aGVHD development, which was associated with a decrease in Th17 cell populations. Our findings suggest a role for rapamycin-mediated autophagy induction as an innovative strategy to optimize the potential of Ad-hMSCs in the treatment of lethal GVHD.

## Methods

### Ethics statement

Ad-hMSCs were obtained by simple liposuction from abdominal subcutaneous fat with informed consent as approved by the Institutional Review Board of Bucheon St. Mary’s Hospital. The procedures for the use of Ad-hMSCs for experimental studies were approved by the Institutional Review Board of Bucheon St. Mary’s Hospital. All experiments were carried out in compliance with the Helsinki Declaration. In addition, all animal experimentation was carried out in strict accordance with the guidelines of the Animal Care and Use Committee of The Catholic University of Korea. The protocol was approved by the Animal Research Ethics Committee of the Catholic University of Korea (permit number: 2012-0155-01), which conformed to all US National Institutes of Health guidelines.

### Culture of human adipose tissue-derived mesenchymal stem cells

Subcutaneous fat was digested with RTase (4 ml/1 g fat; K-STEMCELL Co. Ltd., Seoul, Korea) for 60 min at 37 °C. The digested tissues were filtered through a 100-μm nylon sieve to remove cellular debris. The tissues were then centrifuged at 470 g for 5 min to obtain a pellet. The pellet was resuspended in RCME cell attachment medium (K-STEMCELL Co. Ltd.) and cultured overnight at 37 °C in a humidified atmosphere containing 5 % CO_2_. After 24 h, non-adherent cells were removed and washed with phosphate buffered saline (PBS). The cell medium was changed to RKCM cell growth medium (K-STEMCELL Co. Ltd.) containing 5 % fetal bovine serum (FBS: Invitrogen, Carlsbad, CA, USA). The cells were maintained for four days until confluent (passage zero). When the cells reached 90 % confluency, they were subcultured and expanded in RKCM for two or three passages, at which point they were used for the experiments.

### Animals

Female BALB/c (B/c, H-2k^d^) and C57BL/6 (B6, H-2k^b^) mice, eight to ten weeks of age, were purchased from the Jackson Laboratory (Bar Harbor, ME, USA) and maintained under specific pathogen-free conditions in an animal facility with controlled humidity (55 ± 5 %), light (12 h/12 h light/dark), and temperature (22 ± 1 °C). The air in the facility was passed through a HEPA filtration system designed to exclude bacteria and viruses. Animals were fed mouse chow with ad libitum access to tap water.

### Real-time polymerase chain reaction

Ad-hMSCs were stimulated with rapamycin (50 nM). After incubation for 48 h, mRNA was extracted using RNAzol B (Biotex Laboratories, Houston, TX, USA) according to the manufacturer’s instructions. Reverse transcription of 2 μg total mRNA was performed at 42 °C using the Superscript™ reverse transcription system (Takara, Shiga, Japan). The polymerase chain reaction (PCR) was performed in a 20-μl final volume in capillary tubes in a LightCycler instrument (Roche Diagnostics, Mannheim, Germany). The reaction mixture contained 2 μl LightCycler FastStart DNA MasterMix for SYBR® Green I (Roche Diagnostics), 0.5 μM each primer, 4 mM MgCl_2_, and 2 μl template DNA. All capillaries were sealed, centrifuged at 500 × *g* for 5 s, and then amplified in the LightCycler, with activation of the polymerase (95 °C for 10 min), followed by 45 cycles at 95 °C for 10 s, 60 °C (β-actin) or 59 ~ 60 °C (target) for 10 s, and 72 °C for 10 s (Table 1). The temperature transition rate was 20 °C/s for all steps. The double-stranded PCR product was measured during the 72 °C extension step by detection of fluorescence associated with binding of SYBR Green I to the product. Fluorescence curves were analyzed using LightCycler software v. 3.0 (Roche Diagnostics). The LightCycler was used to quantify the target mRNA. Relative expression levels were calculated as the level of target normalized to that of the endogenous housekeeping gene (β-actin). Melting curve analysis was performed immediately after amplification under the following conditions: 0 s (hold time) at 95 °C, 15 s at 71 °C, and 0 s (hold time) at 95 °C. The rate of temperature change was 20 °C/s, except for 0.1 °C/s in the final step. The melting peak generated represented the amount of specific product amplified. The crossing point (*C*_p_) was defined as the maximum of the second derivative from the fluorescence curve. Negative controls were also included and contained all components of the reaction mixture except template DNA. All samples were processed in duplicate. The following primers were used to amplify human genes: for IL-10, indoleamine 2,3-dioxygenase (IDO); TGF-β, high mobility group box 1 (HMGB1), IL-6, IL-1β, ATG5, LC3A, LC3B, CCR1, CCR2, CCR3, CCR4, CXCR4, CCR7, CCR9, β-actin primer (Table 1).

**Table 1:** Primers used in the polymerase chain reactions

Primer	Forward	Reverse
IDO	5′-TTTGGGTCTTCCCAGAACC-3′	5′-GCGCTGTTGGAAATAGCTTC-3′
TGF-β	5′-TGCGGCAGCTGTACATTGA-3′	5′-TGGTTGTACAGGGCCAGGA-3′
HGF	5′-CCACCATAATCCCCCTCACA-3′	5′-GGCTGGGGCTACACTGGATT-3′
IL-10	5′-CCAAGCCTTGTCTGAGATGA-3′	5′-TGAGGGTCTTCAGGTTCTCC-3′
IL-1β	5′-GGACAAGCTGAGGAAGATGC-3′	5′-TCGTTATCCCATGTGTCGAA-3′
IL-6	5′-AATTCGGTACATCCTCG CGG-3′	5′-GGTTGTTTTCTGCCAGTGCC-3′
HMGB1	5′-GATCCCAATGCACCCAAGAG-3′	5′-TTCGCAACATCACCAATGA-3′
CCR1	5′-ACCATAGGAGGCCAACCCAAAATA-3′	5′-TCCATGCTGTGCCAAGAGTCA-3′
CCR2	5′-CTACCTTCCAGTTCCTCATTTT-3′	5′-ACATTTACAAGTTGCAGTTTTCAG C-3′
CCR3	5′-TTTGTCATCATGGCGGTGTTT TTC-3′	5′-GGTTCATGCAGCAGTGGGAGTAG-3′
CCR4	5′-GAGAAGAAGAACAAGGCGGTGAAGA-3′	5′-GGATTAAGGCAGCAGTGAACAAAAG-3′
CCR7	5′-GCCGAGACCACCACCACCTT-3′	5′-AGTCATTGCATCTGCTCCCTATCC-3′
CCR9	5′-TATACAGCCAAATCAAGGAGGAATC-3′	5′-CATGACCACGAAGGGAAGGAAG-3′
CXCR4	5′-ATCCCT GCCCTCCTGCTGACTATTC-3′	5′-GAGGGCCTTGCGCTTCTGGTG-3′
β - actin	5′-GGACTTCGAGCAAGAGATGG-3′	5′-TGTGTTGGGGTACAGGTCTTTG-3′

### Enzyme-linked immunosorbent assay

In brief, a 96-well plate (Nunc) was coated with 4 μg/ml monoclonal antibodies against IL-10, TGF-beta, IL-6, IL-1beta and HMGB1 (R & D Systems, BD Biosciences, San Jose, CA, USA) at 4 °C overnight. After blocking with phosphate-buffered saline/1 % bovine serum albumin (BSA)/0.05 % Tween 20 for two hours at room temperature (22–25 °C), test samples and the standard recombinant IL-10, TGF-beta, IL-6, IL-1beta and HMGB1 (R & D Systems, BD Biosciences, San Jose, CA, USA) were added to the 96-well plate and incubated at room temperature for two hours. Plates were washed four times with phosphate-buffered saline/Tween 20, and then incubated with 500 ng/ml biotinylated mouse monoclonal antibodies against IL-10, TGF-beta, IL-6, IL-1beta, and HMGB1 (R & D Systems, BD Biosciences, San Jose, CA, USA) for two hours at room temperature. After washing, the plates were incubated with streptavidin–alkaline phosphate–horseradish peroxidase conjugate (Sigma, Sigma-Aldrich, St Louis, MO) for two hours, then washed again and incubated with 1 mg/ml *p*-nitrophenyl phosphate (Sigma, Sigma-Aldrich, St Louis, MO) dissolved in diethanolamine (Sigma, Sigma-Aldrich, St Louis, MO) to develop the color reaction. The reaction was stopped by the addition of 1 M NaOH and the optical density of each well was read at 405 nm. The lower limit of IL-10, TGF-beta, IL-6, IL-1beta, and HMGB1 detection was 10 pg/ml. Recombinant human IL-10, TGF-beta, IL-6, IL-1beta, and HMGB1 diluted in culture medium was used as a calibration standard, ranging from 10 to 2,000 pg/ml. A standard curve was drawn by plotting optical density against the log of the concentration of recombinant cytokines, and used for determination of IL-10, TGF-beta, IL-6, IL-1beta, and HMGB1 concentrations in test samples.

### Confocal microscopic analysis of IL-10 expression

Immunofluorescence staining was performed on the Ad-hMSCs for 48 h with or without rapamycin. Ad-hMSCs were fixed with acetone for 15 min at room temperature and blocked with 10 % goat serum for 30 min at room temperature. After incubation with anti-human IL-10 antibody (Santa Cruz Biotechnology, Dallas, TX, USA), anti-human IDO antibody, and anti–DAPI antibody (R & D Systems Inc., Minneapolis, MN, USA) overnight at 4 °C, the samples were incubated with fluorescein isothiocyanate–conjugated anti-mouse (Santa Cruz Biotechnology) and phycoerythrin-conjugated anti-rabbit IgG secondary antibodies (SouthernBiotech, Birmingham, AL, USA). The stained sections were visualized under a Zeiss confocal microscope (LSM 510 META) at 200× and 400× magnification.

### Mixed lymphocyte reaction and suppression assay

The mixed lymphocyte reactions (MLR) consisted of 5 × 10^4^ murine CD4^+^ effector cells stimulated with anti-CD3 (1 μg/ml), anti-CD28 (1 μg/ml), IL-1β (20 ng/ml), IL-6 (20 ng/ml), and IL-23 (20 ng/ml) to induce Th17 cells and 5 × 10^4^ irradiated (40 Gy) allogeneic murine peripheral blood mononuclear cells (PBMCs) in round-bottomed 96-well plates (Nunc, Roskilde, Denmark) with PBMC culture medium (PCM) consisting of MEM-a supplemented with 2 mM L-glutamine, 1 % penicillin/streptomycin (P/S) and 10 % heat-inactivated human serum. Suppression assays were performed to determine the immunomodulatory capacities of Ad-hMSCs (various concentrations), Ad-hMSCs (1:10) and rapamycin treated-Ad-hMSCs (1:10) (alul ratios: indicated cells/effector cells) in the MLR. CD4^+^ T cell proliferation was investigated by adding ^3^H-thymidine (1 μCi/well; GE Healthcare, Amersham, Buckinghamshire, UK) to the culture medium and incubating for 8 h. The level of ^3^H-thymidine incorporation was measured using a Beckman liquid-scintillation counter (Beckman Coulter Inc., Brea, CA, USA).

### Flow cytometry analysis

Monoclonal antibodies (mAbs) conjugated to fluorescent dyes targeting human CD13, CD90, CD105, CD29, CD44, CD11b, CD19, CD31, CD34, CD45, CD25, and HLA-DR (BD Biosciences, Schwechat, Austria) were used to characterize the MSCs. Their surfaces were stained using different combinations of the following mAbs: CD13-PE (L138, IgG_1_, κ, Pharmingen, San Diego, CA, USA), CD90-PE (5E10, IgG1, κ Pharmingen), CD11b-PE (ICRF44, IgG_1_, κ, Pharmingen), CD19-PE (HIB19, IgG1, κ, Pharmingen ), CD34-PE (8G12, IgG1, κ, Pharmingen ), CD34-FITC (MMA, IgM, Pharmingen ), CD25-APC (PC61, IgG_1_, λ, Pharmingen), CD105-APC (RPA-T4, IgG1; BioLegend, San Diego, CA, USA), CD29-PE (RPA-T8, IgG1, κ; Pharmingen), CD44-FITC (HI100, IgG2b, κ; Pharmingen), CD45-APC (M-A251, IgG1, κ; Pharmingen), and HLA-DR-PE (G46-6, IgG2a, κ; Pharmingen). For staining intracellularly, the cells were washed, fixed, permeabilized, and incubated with mAbs against IL-17 (PE, eBio64dec17, IgG1, κ; eBioscience, San Diego, CA, USA), IFN-γ (FITC, 4S.B3, IgG1, κ; eBioscience; and PE, B27, IgG1, κ; Pharmingen), IL-4 (APC, MP4-25D2, IgG1, κ; eBioscience), and Foxp3 (FITC, PCH101, IgG2a, κ; eBioscience). Appropriate isotype controls were used for gating purposes. Cells (1 × 10^5^ cells/well) were analyzed using a FACSCalibur flow cytometer (BD Biosciences). The data were analyzed using the FlowJo software (Tree Star, Ashland, OR, USA).

### Transmission electron microscopy

The cells were harvested, centrifuged (215 × g for 10 min), washed with cold PBS and fixed with 2.5 % glutaraldehyde (in 0.2 M sodium cacodylate, pH 7.4). The samples were then fixed in 1 % OsO4 for 1 h at 4 °C, dehydrated with increasing concentrations of ethanol, embedded in spurr resin and sectioned. The ultrathin sections were stained with uranyl acetate and observed by transmission electron microscopy (TEM).

### Western blot analysis

To examine the effects of rapamycin, Ad-hMSCs were incubated for 48 h in the presence of rapamycin. The membrane was incubated overnight at 4 °C with primary antibodies against the following: p-mTOR, mTOR, p-Rictor, Rictor, p-Raptor, Raptor, Beclin1, ATG5, ATG7, LC3 II (all antibodies were from Cell Signaling Technology Inc., Danvers, MA, USA), and β-actin (Sigma, Sigma-Aldrich, St Louis, MO). After washing in Tris-buffered saline and Tween 20 (TTBS), the reactive bands were visualized using an ECL detection kit and Hyperfilm-ECL reagents (Amersham Pharmacia Biotech, Little Chalfont, Bucks, UK).

### Chemotaxis assay

Cell migration was evaluated using a 48-well modified Boyden chamber as previously described, with minor modifications [29]. The polycarbonate filter (12-μm pore size, CN110416; Neuroprobe, Bethesda, MD, USA) was pre-coated with fibronectin (5 μg/mL; Sigma); Ad-hMSCs were resuspended at 5 × 10^5^/mL in the appropriate medium supplemented with 1 % FBS and seeded in the upper chamber. CD4^+^ T cells were able to attract human MSCs in a classical chemotaxis assay [30]. CD4^+^ T cells were used as chemoattractants in the lower compartment. The chambers were incubated overnight at 37 °C. Results are expressed as the mean number of net migrated cells relative to the control cells counted in 10 microscope fields at high-power magnification (×1,000). Each experiment was performed in triplicate.

### The bone marrow transplantation model

The BMT procedure was performed as described previously [31]. To induce aGVHD, recipients (B/c mice) were injected intravenously with 5 × 10^6^ donor (B6) bone marrow cells and 1 × 10^7^ donor splenocytes (as a source of allogeneic T cells) after lethal irradiation with 800 cGy [32]. To induce the syngeneic BMT model, recipient B/c mice were injected intravenously with 5 × 10^6^ donor (B/c) bone marrow cells and 1 × 10^7^ donor B/c splenocytes after lethal irradiation with 800 cGy. All experiments were conducted with 12 mice per group. The recipient mice were divided randomly into the control group and the therapy (Ad-hMSCs or rapamycin-treated Ad-hMSCs) groups. One day after GVHD induction, the mice were administered saline (control group), Ad-hMSCs or rapamycin (50 nM)-treated Ad-hMSCs (1 × 10^6^) via the tail vein. Survival after BMT was monitored daily, and the day of death was recorded as that on which the mouse died spontaneously. The extent of clinical GVHD was assessed twice per day for 30 days after the BMT, using a scoring system that summed the changes over five clinical parameters: weight loss, posture, activity, fur texture, and skin integrity. Animals were sacrificed humanely when they exhibited the euthanasia GVHD criteria (>20 % weight loss or animals that received a score ≥ 6.5).

### Histopathologic analysis of GVHD target organs

The liver and skin tissues isolated from the mice in each group (control, Ad-hMSC or rapamycin-treated-Ad-hMSC) were fixed in 10 % formalin, embedded in paraffin, sectioned at 7-μm thickness, deparaffinized using xylene, dehydrated through an alcohol gradient and stained with hematoxylin and eosin (H & E). GVHD was scored by two trained pathologists, who were blinded to the treatment groups, according to previously published histopathology scoring systems [33].

### Staining for confocal microscopic analysis

Spleen tissues were obtained 14 days after BMT, snap-frozen in liquid nitrogen, and stored at −80 °C. Tissue cryosections (7-μm thickness) were fixed in 4 % (v/v) paraformaldehyde and stained using fluorescein isothiocyanate (FITC)-, phycoerythrin (PE)-, PerCP-Cy5.5-, or allophycocyanin (APC)-conjugated monoclonal antibodies to mouse CD4, interferon-gamma (IFN-γ), IL-4, IL-17, CD25 or Foxp3 (eBioscience). After incubation overnight at 4 °C, stained sections were visualized by confocal microscopy (LSM 510 Meta; Zeiss, Göttingen, Germany). Positive cells were counted manually at a higher magnification (projected on a screen) by four individuals, and the results were expressed as means ± SD.

### Statistical analysis

Statistical analysis was performed using IBM SPSS Statistics 20 for Windows (IBM Corp., Armonk, NY, USA). One-way analysis of variance followed by Bonferroni’s *post-hoc* test was used to compare the differences between three or more groups. Comparisons of numerical data between two groups were performed by Student’s t-test or a nonparametric Mann–Whitney U test. The Mann–Whitney U test was used for statistical analysis of the clinical scores, while the Mantel-Cox log-rank test was used to analyze the survival data. The data were considered significantly different at *P* < 0.05.

## Results

### Characterization of rapamycin-treated Ad-hMSCs

**Fig. 1:**
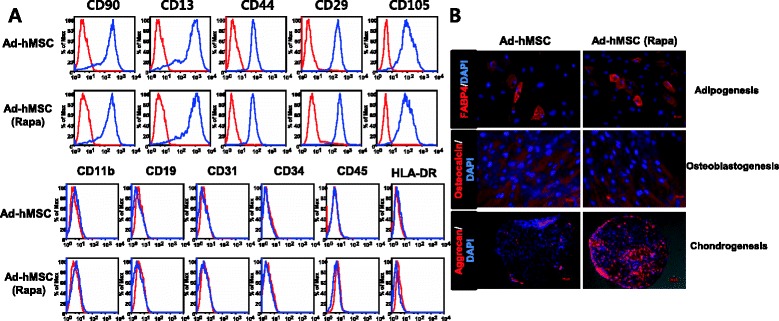
Surface markers and multilineage differentiation capacity of rapamycin-treated and untreated Ad-hMSCs. **a** Expression of markers in Ad-hMSCs was analyzed by flow cytometry. One representative analysis is reported. From left to right: CD90, CD13, CD44, CD29, CD105 (*upper row*); CD11b, CD19, CD31, CD34, CD45, HLA-DR (*lower row*). Red histograms, cells stained with isotype control; blue histogram, cells isolated with specific antibodies. **b** MSCs were plated on each differentiation medium. After 1 week (adipogenic differentiation) or 3 weeks (osteoblastogenic and chondrogenic differentiation), cells were fixed and stained with FABP-4 (*top*), osteocalcin (*middle*), or aggrecan (*bottom*) and visualized by fluorescence microscopy. Representative images of MSC-induced differentiation into adipogenic, osteogenic, and chondrogenic pathways.. *Ad-hMSCs* adipose tissue-derived human MSCs, *MSCs* mesenchymal stem cells, *HLA-DR* human leukocyte antigen-DR

Flow cytometry analysis was used to assess whether rapamycin-treated and untreated Ad-hMSCs showed typical MSC surface marker profiles. The Ad-hMSCs in both groups expressed the mesenchymal cell surface markers CD90, CD13, CD44, CD29, and CD105, and were negative for CD11b, CD19, CD31, CD34, CD45, and HLA-DR (Fig. 1a). It was noted that rapamycin-treated Ad-hMSCs had a very similar surface antigen profile to that of untreated Ad-hMSCs. To determine whether rapamycin pre-treatment in Ad-hMSCs can affect the cells’ capability to differentiate into multiple cell lineages [34], Ad-hMSCs were cultured under different conditions: adipogenic medium (Fig. 1b, upper panel), osteogenic medium (Fig. 1b, medium channel), or chondrogenic medium (Fig. 1b, lower panel). The treatment concentration of rapamycin (50 nM) did not show cellular toxicity, as assessed with MTT assay (data not shown).

### Rapamycin enhanced the immunoregulatory properties of Ad-hMSCs

**Fig. 2:**
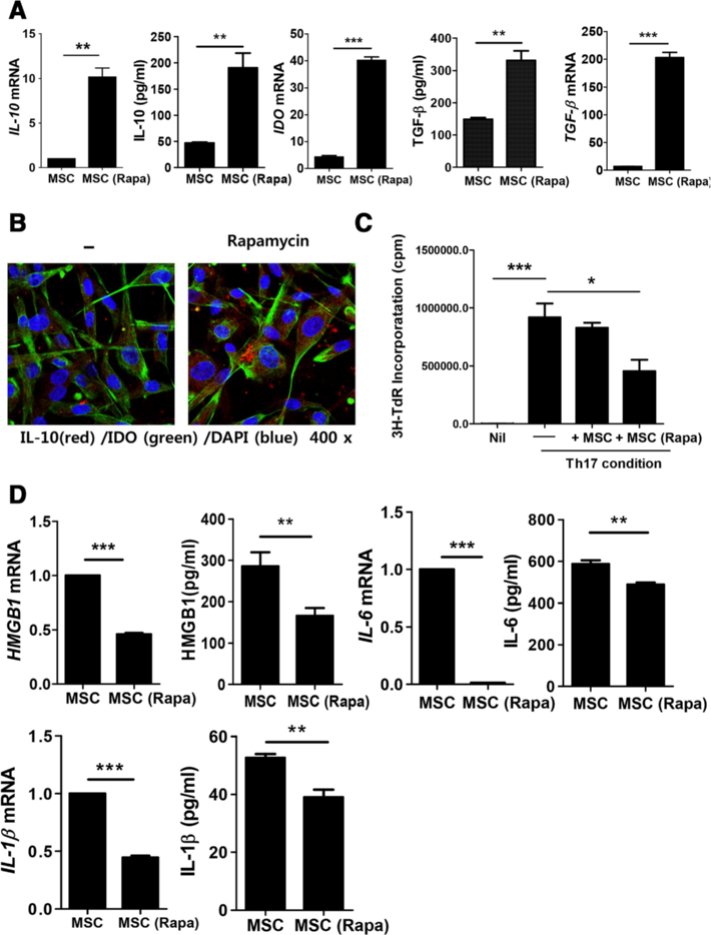
Immunoregulatory properties of human adipose tissue-derived mesenchymal stem cells (Ad-hMSCs) by rapamycin treatment. **a** Expression of IL-10, IDO, and TGF-β was analyzed by real-time PCR and sandwich ELISA in Ad-hMSCs with or without rapamycin. **b** Immunofluorescence staining of cultured Ad-hMSCs treated with or without rapamycin with IL-10 (red), IDO (green) and counterstained with DAPI (blue). **c** T cell proliferation under Th17 polarizing conditions by rapamycin-treated Ad-hMSCs and untreated Ad-hMSCs. **d** Expression of HMGB1, IL-6, and IL-1β was analyzed by real-time PCR and sandwich ELISA in Ad-hMSCs with or without rapamycin. Data are presented as mean ± SD (standard deviation) and represent three independent experiments (*n* = 3). ^*^ *P* < 0.05; *** *P* < 0.001

To assess whether rapamycin treatment influenced the immunoregulatory activities in Ad-hMSCs, the mRNA synthesis of IL-10, IDO, and TGF-β were analyzed by real-time PCR in Ad-hMSCs with or without rapamycin. IL-10, IDO, and TGF-β mRNA levels were increased by rapamycin pretreatment. Also, the concentrations of IL-10 and TGF-β in culture supernatants measured by ELISA were also increased by rapamycin pretreatment (Fig. 2a).

IL-10 expression was compared between untreated Ad-hMSCs and rapamycin-treated Ad-hMSCs by confocal microscopy using triple-immunofluorescence staining. IL-10 (red) and IDO (green) expression was detected abundantly in the rapamycin-treated Ad-hMSCs, whereas expression was faint in the Ad-hMSCs without rapamycin treatment (Fig. 2b). To ascertain whether rapamycin could augment MSC-induced immunoregulation, rapamycin-treated Ad-hMSCs were cultured for 48 h in the presence or absence of rapamycin (Fig. 2c). In vitro co-culture studies revealed that rapamycin-treated Ad-hMSCs could suppress clonal expansion of Th17 cells more effectively than untreated Ad-hMSCs (Fig. 2c). Next, to determine whether rapamycin regulated the molecules known to be strongly associated with the development of Th17 cells in Ad-hMSCs, protein and mRNA expression levels of high-morbidity group box chromosomal protein 1 (HMGB1) [35], IL-6 [36], and IL-1β [37] were quantified by sandwich ELISA and real-time PCR, respectively. The results showed significantly suppressed levels of HMGB1-, IL-6-, and IL-1β mRNA and lower concentrations of these molecules in the culture supernatants of rapamycin-treated Ad-hMSCs compared with untreated Ad-hMSCs (Fig. 2d).

### Rapamycin induced autophagy in Ad-hMSCs

**Fig. 3:**
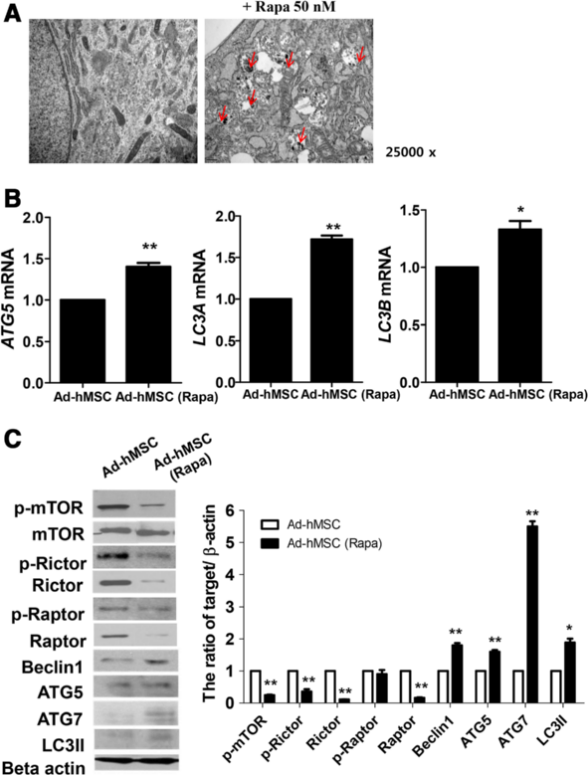
Rapamycin induced autophagy in Ad-hMSCs. **a** Transmission electron microscopy verified that Ad-hMSCs treated with rapamycin exhibited numerous cytoplasmic vacuoles, most of which were identified clearly as autophagosomes (with a visible double membrane; *red arrow*). **b** mRNA expression of autophagy markers in Ad-hMSCs treated with or without rapamycin was detected by real-time PCR. **c** Protein extracts from Ad-hMSCs treated with or without rapamycin were analyzed by Western blotting using antibodies targeting phosphor-mTOR, mTOR, phosphor-Rictor, Rictor (a novel binding partner of mTOR), phosphor-Raptor, Raptor, Beclin 1, ATG5, ATG7, and LC3II (*left panel*). Data in the graphs show the relative intensities of the target proteins normalized to β-actin; values are presented as means ± SD (*n* = 3). ^*^ *P* < 0.05; ^**^ *P* < 0.01. *Ad-hMSCs* adipose tissue-derived human mesenchymal stem cells

To address the question of how rapamycin affected the immunoregulatory properties of Ad-hMSCs, Ad-hMSCs were incubated with or without 50 nM rapamycin. In comparison with untreated cells, rapamycin induced the formation of small vacuoles inside the cells after 48 h incubation, consistent with its autophagy-inducing properties (Fig. 3a). The TEM revealed that Ad-hMSC exposure to rapamycin resulted in the appearance of autophagic vacuoles (AV) (Fig. 3a). Beclin 1, ATG5, ATG7, and LC3 play a central role in autophagy and are common indicators of the induction of autophagy [38–40]. Normally, LC3 is present in the cytosol diffusely, but it is converted from LC3-I (18 kD) to LC3-II (16 kD) upon autophagy and accumulates on the autophagosome membrane, appearing as dots on the membrane [41, 42]. In this study, the expression of markers associated with the autophagy pathway in Ad-hMSCs treated with or without rapamycin were analyzed. The expression of mRNA for ATG5, LC3A, and LC3B was determined using real-time PCR. The expression of ATG5, LC3A, and LC3B mRNA was significantly increased in the rapamycin-treated Ad-hMSCs compared with the untreated Ad-hMSCs (Fig. 3b). To identify the molecular mechanisms modulated by rapamycin, we investigated whether rapamycin interfered directly with mTOR and autophagy signaling. Western blotting showed that rapamycin not only provoked the expression of both Beclin 1 and LC3II but also suppressed the expression of mTOR and mTOR protein complexes (Rictor and Raptor) (Fig. 3c). These data together proved that rapamycin treatment in Ad-hMSCs induced autophagy and was associated with inhibition of mTOR/Rictor/Raptor signaling.

### Rapamycin increased the migration capacity and expression of chemokine receptors in Ad-hMSCs

**Fig. 4:**
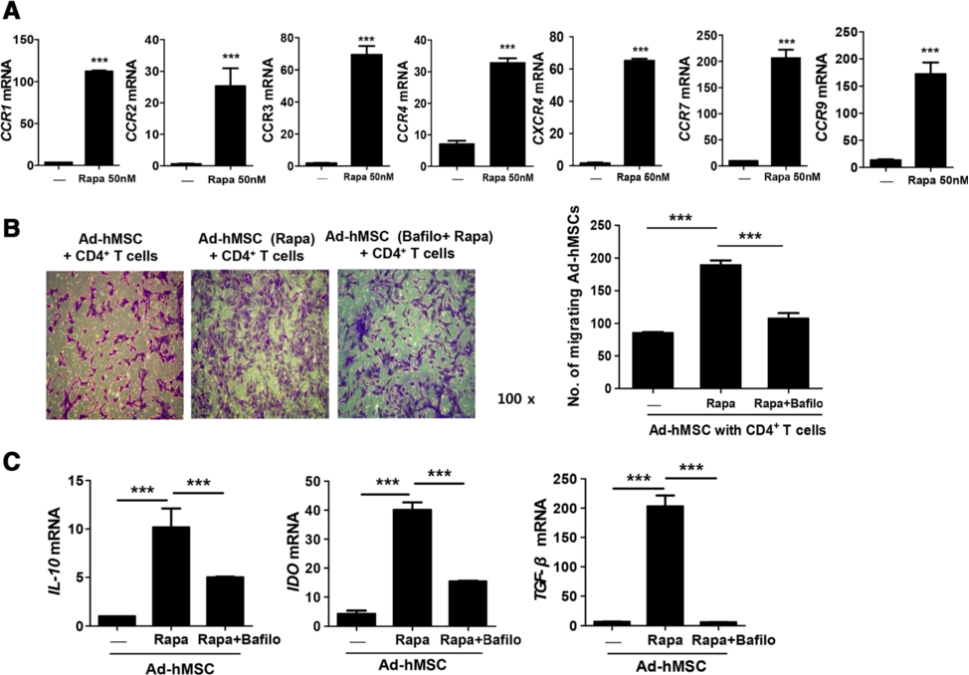
Migration capacity of Ad-hMSCs is improved by rapamycin (Rapa) via increased expression of chemokine receptors. **a** mRNA expression of chemokine receptors in Ad-hMSCs treated with or without rapamycin. Surface expression of CCR1, CCR2, CCR3, CCR4, CCR7, CCR9, and CXCR4 on Ad-hMSCs was assessed by real-time PCR. Graphs represent the mean chemokine receptor expression of Ad-hMSCs treated with rapamycin (50 nM) compared with the negative control (untreated Ad-hMSCs), and bars represent the standard deviation (SD) from three independent experiments (*n* = 3). **b** Migration assay of Ad-hMSCs in response to CD4^+^ T cells. Two thousand Ad-hMSCs either untreated (*left*), treated with rapamycin (*middle*), or treated with bafilomycin (Bafilo) and Rapa (*right*) were placed in the top well of a chemotaxis chamber, with CD4^+^ T cells present in the bottom well. After 2 h, the migrating cells were counted. Representative images show the migrating Ad-hMSCs (*upper panel*). The bar graphs represent the numbers of migrating Ad-hMSCs as means ± SD (*lower panel*). **c** mRNA expression of IL-10 (*left*), IDO (*middle*), and TGF-β (*right*) of Ad-hMSCs was assessed by real-time PCR. Graphs represent the mean mRNA expression of Ad-hMSCs either untreated, treated with Rapa, or treated with Rapa and Bafilo. Error bar represents the SD from independent experiments (n = 3). ^***^ *P* < 0.001. *Ad-hMSCs* adipose tissue-derived human mesenchymal stem cells

To evaluate the in vitro migration capacity of rapamycin-treated Ad-hMSCs, the mRNA expression of chemokine and chemokine receptors was investigated by real-time PCR. Ad-hMSCs expressed C-C chemokine receptor type 1 (CCR1), 2 (CCR2), 3 (CCR3), 4 (CCR4), 7 (CCR7), and 9 (CCR9) and C-X-C chemokine receptor type 4 (CXCR4). The expression of CCR1, CCR2, CCR3, CCR4, CCR7, CCR9, and CXCR4 mRNA was significantly increased in the rapamycin-treated Ad-hMSCs compared with the untreated Ad-hMSCs (Fig. 4a). As migration of MSCs from circulation into injured tissues can contribute to their immunoregulatory capacities in inflammatory conditions, we investigated whether their migration efficiency is influenced by rapamycin treatment. The in vitro migration efficiency of rapamycin-treated Ad-hMSCs towards the CD4^+^ T cells was increased significantly, with a 2.7-fold increase compared with the untreated cells (Fig. 4b). Interestingly, the enhanced migration capacity seen in the rapamycin-treated Ad-hMSCs was reversed significantly by bafilomycin (an autophagy inhibitor) (Fig. 4b). Next, to determine whether the immunoregulatory molecules produced by MSCs might be affected by autophagy, the mRNA level of IL-10, IDO, and TGF-β was analyzed in rapamycin-treated Ad-hMSCs in the presence or absence of bafilomycin. The enhanced mRNA expression of these molecules by rapamycin pretreatment in Ad-hMSCs was markedly reversed by bafilomycin (Fig. 4c). Taken together, autophagy-induction properties of rapamycin in Ad-hMSCs contributed to the enhanced migration of, and intensified expression of, immunoregulatory molecules.

### Rapamycin-treated Ad-hMSCs protect mice from aGVHD after bone marrow transplantation

**Fig. 5:**
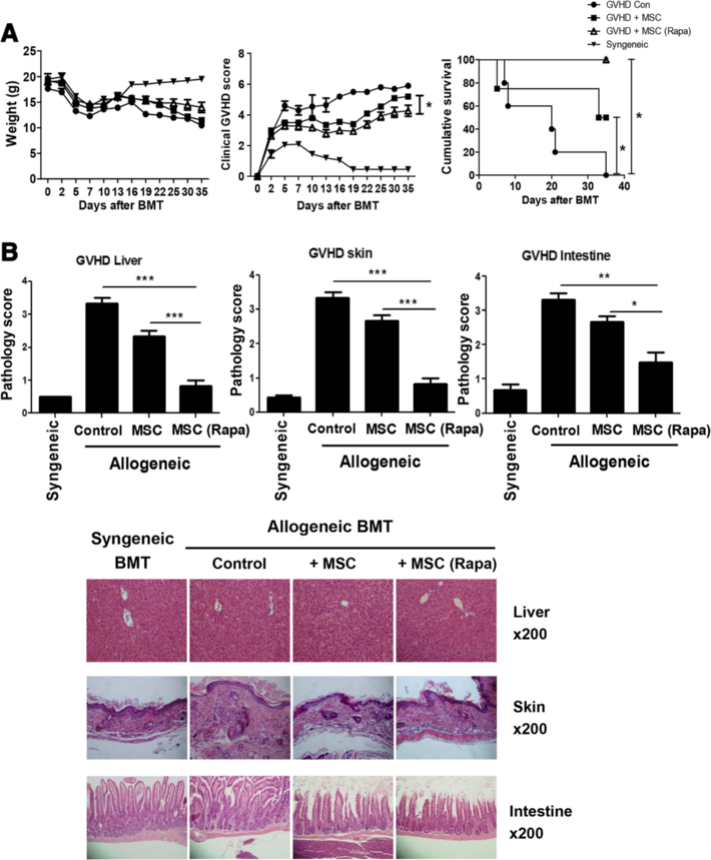
The inhibitory capacity of Ad-hMSCs on aGVHD severity was augmented by rapamycin treatment. **a** Recipient BALB/c (B/c) mice received 5 × 10^6^ bone marrow cells and 1 × 10^7^ splenocytes from B6 (allogeneic) or B/c mice (syngeneic) after lethal irradiation with 800 cGy. On day 1 post BMT, allogeneic BMT recipient mice received intravenous administration of Ad-hMSCs, rapamycin-treated Ad-hMSCs, or saline (control). Mice with aGVHD were monitored for weight, clinical signs, and the ability to survive. Combined data from two independent experiments (*n* = 12 per group) are shown. Improvements in the clinical aGVHD score after allogeneic BMT was assessed in terms of (reduced) weight loss, posture, activity, fur texture, and skin integrity. ^*^ *P* < 0.05 (**b**) Pathology scores (*upper panel*) and histopathology of the liver, skin, and intestine after BMT (*n* = 12 per group); the photographs were taken from one of two independent experiments. The sections were stained with H & E (original magnification × 200). The *upper panel* shows the mean scores of the liver, skin, and intestine of four groups. Results are expressed as means ± SD. ^*^ *P* < 0.05; ^**^ *P* < 0.01; ^***^ *P* < 0.001. *Ad-hMSCs* adipose tissue-derived human mesenchymal stem cells, *aGVHD* acute graft-versus-host disease, *BMT* bone marrow transplantation

Because rapamycin significantly enhanced the immunoregulatory properties of Ad-hMSCs, we assessed its effects in vivo using a murine model of aGVHD. Severe aGVHD occurred in all B/c (H-2k^d^) recipient mice undergoing BMT and infusion of donor (B6 mice, H-2k^b^) splenocytes. One day after GVHD induction, the mice were administered control (saline), Ad-hMSCs, or rapamycin-treated Ad-hMSCs via the tail vein. Acute GVHD animals transplanted with Ad-hMSCs showed reduced weight loss, less severe clinical scores of aGVHD, and significantly delayed aGVHD-induced lethality compared with the control aGVHD mice (Fig. 5a). The administration of rapamycin-treated Ad-hMSCs in the recipient mice showed less severe clinical scores of aGVHD compared with untreated-Ad-hMSCs. Furthermore, all the recipient mice treated with rapamycin-treated Ad-hMSCs survived for 40 days after BMT (Fig. 5a). Acute GVHD targets the liver, skin, and intestine. Mice from each treatment group were sacrificed on day 12 post-BMT after GVHD-induction. To determine the protective effects of the rapamycin-treated Ad-hMSCs on the development of aGVHD, the tissue pathologies of the liver, skin, and intestine were evaluated. Recipient mice administered rapamycin-treated Ad-hMSCs showed less severe aGVHD pathology scores for the liver, skin, and intestine, whereas recipient mice administered untreated Ad-hMSCs showed moderate aGVHD (Fig. 5b). The histopathologic changes in the nucleus and cytoplasm, as well as the extent of infiltrating inflammatory cells found in the aGVHD target organs were reduced dramatically in the mice administered rapamycin-treated Ad-hMSCs (Fig. 5b).

### Rapamycin treatment in Ad-hMSCs can modulate the differentiation of effector T cells and the production of inflammatory cytokines in vivo

**Fig. 6:**
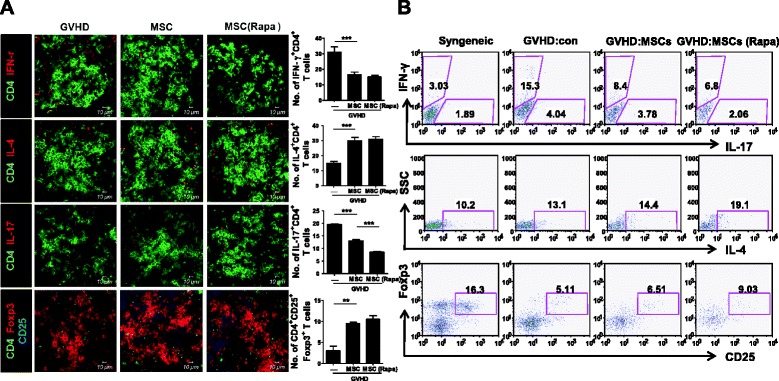
Analysis of T helper cell subsets in aGVHD mice administrated Ad-hMSCs or rapamycin-treated Ad-hMSCs. **a** Recipient BALB/c mice received 5 × 10^6^ bone marrow cells and 1 × 10^7^ splenocytes from B6 mice after lethal irradiation with 800 cGy. On day 1 post BMT, recipient mice received intravenous administration of Ad-hMSCs, rapamycin-treated Ad-hMSCs, or saline (control). Intracellular cytokine levels were determined in splenic CD4^+^ T cells by confocal microscopy on day 12 after BMT. Histopathology and confocal microscopic images were obtained for each mouse (*n* = 5), and representative images are shown. Bars represent the means ± SDs of data from five mice per group. ^**^ *P* < 0.01; ^***^ *P* < 0.001. The data are representative of at least three independent experiments. **b** The proportion of IFN-γ^+^ (mainly Th1), IL-4^+^ (mainly Th2), IL-17^+^ (mainly Th17), and CD25^+^Foxp3^+^ (mainly Treg) among splenic CD4^+^ T cells were assessed ex vivo by intracellular fluorescence-activated cell sorting. *aGVHD* acute graft-versus-host disease, *Ad-hMSCs* adipose tissue-derived human mesenchymal stem cells, *BMT* bone marrow transplantation

T cells are absolutely essential to cause GVHD [43]. Therefore, to investigate the in vivo mechanism of rapamycin-treated Ad-hMSCs in the aGVHD murine model, the numbers of CD4^+^IFN-γ^+^, CD4^+^IL-4^+^, CD4^+^IL-17^+^, and CD4^+^CD25^+^Foxp3^+^ T cells in spleens isolated from each group were counted after confocal staining. The numbers of CD4^+^IFN-γ^+^ (mainly Th1) and CD4^+^IL-17^+^ T (mainly Th17) cells in the spleens were lower in the Ad-hMSC-administered GVHD mice compared with the control GVHD mice. On the other hand, the numbers of CD4^+^IL-4^+^ (mainly Th2) and CD4^+^CD25^+^Foxp3^+^ T cells (mainly regulatory T [Treg] cells) were higher in the Ad-hMSC-administered group (Fig. 6a and b). For our interest, recipient mice receiving rapamycin-treated Ad-hMSCs showed a significantly decreased population of Th17 cells in vivo compared to those receiving untreated Ad-hMSCs (Fig. 6a and b). In summary, administration of rapamycin-treated Ad-hMSCs had a greater suppressive potential on the development of aGVHD than did untreated Ad-hMSCs. The therapeutic potential of rapamycin-treated Ad-hMSCs in an animal model of aGVHD might be achieved by enhanced migration capacity and optimized immunoregulatory potential of the cells. Furthermore, the autophagy-inducing properties of rapamycin could play a critical role in the optimization of the use of Ad-hMSCs to prevent the development of lethal aGVHD.

## Discussion

We demonstrated that rapamycin, an mTOR inhibitor, optimized the immunoregulatory properties of Ad-hMSCs without affecting the panel of MSC markers evaluated (positive for CD105, CD29, and CD44 and negative for CD45 and HLA-DR). The enhanced immunoregulatory potential induced by rapamycin was associated with induction of autophagy in Ad-hMSCs and decreased mTOR/Rictor/Raptor activity. Rapamycin treatment in Ad-hMSCs also increased their migration potential significantly. Ultimately, the administration of rapamycin-treated Ad-hMSCs led to reduced mortality in aGVHD recipients and attenuated the clinical severity of aGVHD.

One previous study demonstrated that systemic (intraperitoneal) treatment with rapamycin as an adjunct therapy with MSC infusion in a mouse model of xenogenic GVHD resulted in a significantly decreased mortality rate, whereas there was no clinical benefit in MSC alone-treated GVHD mice compared with the control group [44], suggesting the beneficial effects of rapamycin in the setting of MSC infusion. Based on the immunosuppressive and antitumor properties of rapamycin, it is being increasingly used in GVHD prophylaxis [45] and steroid-refractory GVHD [46]. Despite its significant advantages, systemic administration of rapamycin showed specific adverse events including reversible cytopenia that seems to be dose-dependent and hyperlipidemia, occurring in 40 % and 61 % of organ transplant recipients [47–49], raising concerns about its clinical uses in GVHD patients. Since the first report that described the efficacy of MSCs for the treatment of aGVHD [9], many publications have presented a broad spectrum of response rate varying from 0 % [50] to 80 % [51]. In some cases, MSCs resulted in accelerated graft rejection, even coadministered with cyclosporine [52]. However, the clinical application of MSCs is increasingly suggested as a third line treatment option in the steroid-refractory aGVHD and may have a greater role in the future [53], although this approach to prevent or treat aGVHD is still far from routine. Thus, to optimize the immunoregulatory properties of MSCs is now warranted. Until now, there have been several attempts to optimize the immunoregulatory actions of MSCs to maximize their therapeutic potential, such as mRNA transfection [54], adjunctive therapy [55], and selection of infusion route [56]. Compared to these approaches, it is much easier to adapt our work which showed that simple exposure to a low concentration of rapamycin (nanomolar level) can attain optimized immunoregulatory potential of Ad-hMSCs.

Acute GVHD is an immunological disorder that mainly affects the skin, gastrointestinal tract, liver and lungs, leading to tissue destruction. Classically, the interactions between host APCs and donor T cells are reported to activate the T helper 1 (Th1) cell-driven inflammatory process, leading to development and progression of aGVHD. However, emerging new data indicate that Th17 cells and Th17-associated cytokines play a central role in the occurrence of aGVHD [57, 58]. IL-17 contributed to the development of aGVHD in recipient mice by recruiting or priming Th1 cells during the early stages of the disease [59], reflecting a shift from Th1 to Th17 cells in the physiopathology of aGVHD. In contrast to the Th17 subset, CD4^+^CD25^+^Foxp3^+^ Treg cells play a key role in the control of alloreactive responses and in the maintenance of immune tolerance after allogeneic BMT.

Treg cells that are critical in immune tolerance to alloantigen can prevent GVHD and show a suppressive role in transplant tolerance [60], suggesting a therapeutic potential in GVHD prevention. Indeed, CD4^+^CD25^+^ Treg cells expanded *ex vivo* have been shown to delay or even prevent the severity of GVHD in an animal model [61, 62]. Several preclinical studies have demonstrated that CD4^+^CD25^+^Foxp3^+^ Treg cells play critical roles for the induction and maintenance of tolerance to alloantigens via a costimulatory pathway [62, 63], suggesting the emergence of a new modality of cellular therapy to prevent the development of lethal GVHD. Furthermore, Sakaguchi et al. found that CD4^+^CD25^+^ Treg cells can attenuate the rejection of allogeneic skin grafts [64]. In line with these findings, the fraction of CD4^+^Foxp3^+^ Treg cells was relatively small in human GVHD [65]. The deficiency of Treg cells may be responsible for the consequent pathological differentiation of effector CD4^+^ T cell subpopulations after BMT, in that there could be a relative shift from Treg to Th1/Th17 lineages. In a phase I clinical trial, the incidence of grade 2–4 aGVHD in patients treated with ex-vivo expanded Treg infusion was 43 % compared with 61 % in historical controls who were identically treated [66].

Here, we have shown that rapamycin can induce autophagy of Ad-hMSCs, while preserving the expression of surface markers and multilineage differentiation potentials of MSCs under in vitro conditions. That may underlie the effective immunoregulatory property of Ad-hMSCs by rapamycin treatment that was shown in a murine model of aGVHD.

Prior to the clinical use of MSCs, it is important to understand their biological roles fully. MSCs produce immunosuppressive molecules and various growth factors that facilitate tissue repair [67]. Inflammatory mediators including cytokines, such as TNF-α and IFN-γ, can stimulate the release of many growth factors by MSCs, promoting tissue regeneration and repair [68]. Together with their reparative functions, MSCs retain their immunosuppressive functions, e.g., by inducing IL-10-secreting macrophages [69]. GVHD manifests as a “cytokine storm”, the result of a BMT conditioning regimen, activation of donor T cells by recipient APCs, and the subsequent damage of host tissues by large numbers of activated T cells (effector stage). As MSCs produce growth factors and cytokines, issues with the therapeutic use of MSCs in GVHD include minimization of the production of proinflammatory and pro-angiogenic molecules and optimization of MSC delivery to the target tissues. In this study, the focus was on the optimization strategy for MSCs with regard to their migration capacity and the production of inflammatory molecules. In addition, a strategy to optimize the potential of MSC-based cell therapy to prevent lethal GVHD may reduce the noted adverse events of the rapamycin than those taken systemically. To summarize, our findings indicated that rapamycin pretreatment of MSCs upregulated their immunoregulatory properties and downregulated the production of proinflammatory molecules involved in Th17 differentiation, without affecting MSC characterization. These effects were associated with autophagy induction, and rapamycin treatment enhanced the migratory potential of MSCs. Encouragingly, rapamycin-treated MSCs exerted more effective immunosuppressive properties in a murine model of aGVHD. Therefore, autophagy induction by rapamycin may represent an innovative strategy in the optimization of the use of MSCs in other T-cell mediated inflammatory disorders.

## Conclusions

This study demonstrated that rapamycin treatment of Ad-hMSCs can optimize their immunomodulatory properties without affecting MSC characterization. The enhanced immunoregulatory property of Ad-hMSCs by rapamycin treatment is associated with inhibition of mTOR/Rictor/Raptor signaling. The in vitro migration efficiency of rapamycin-treated Ad-hMSCs towards CD4^+^ T cells is increased compared with the untreated cells. The autophagy induction property of rapamycin in Ad-hMSCs, at least partially, contributes to the enhanced migration efficiency. In a murine model of aGVHD, recipient mice receiving rapamycin-treated Ad-hMSCs show less severe clinical scores of aGVHD compared with those receiving untreated-Ad-hMSCs. The greater suppressive potential of rapamycin-treated Ad-hMSCs shown in vivo is associated with decreased Th17 subset. Our finding has opened up a new way of optimization of MSCs against the development of lethal aGVHD.

## Abbreviations

Ad-hMSCs: Adipose tissue-derived human MSCs
aGVHD: Acute graft-versus-host disease
B/c: BALB/c
B6: C57BL/6
BM-MSCs: Bone marrow- derived mesenchymal stem cells
BMT: Bone marrow transplantation
CCR: C-C chemokine receptor
CXCR: C-X-C chemokine receptor
FBS: Fetal bovine serum
GVHD: Graft-versus-host disease
H &E: Hematoxylin and eosin
HMGB1: High mobility group box 1
IDO: Indoleamine 2,3- dioxygenase
mAbs: Monoclonal antibodies
MLR: Mixed lymphocyte reaction
PBMCs: Peripheral blood mononuclear cells
PCR: Polymerase chain reaction
TEM: Transmission electron microscopy
Th17: Interleukin-17-producing CD4^+^ T
Treg: Regulatory T
