# Erratum to: Optimization of adipose tissue-derived mesenchymal stem cells by rapamycin in a murine model of acute graft-versus-host disease

**DOI:** 10.1186/s13287-016-0336-x

**Published:** 2016-05-31

**Authors:** Kyoung-Woon Kim, Su-Jin Moon, Min-Jung Park, Bo-Mi Kim, Eun-Kyung Kim, Sung-Hee Lee, Eun-Jung Lee, Byung-Ha Chung, Chul-Woo Yang, Mi-La Cho

**Affiliations:** Convergent Research Consortium for Immunologic disease, Transplant Research Center, The Catholic University of Korea, Seoul, South Korea; Division of Rheumatology, Department of Internal Medicine, College of Medicine, The Catholic University of Korea, Seoul, South Korea; The Rheumatism Research Center, The Catholic University of Korea, Seoul, South Korea; Division of Nephrology, Department of Internal Medicine, College of Medicine, The Catholic University of Korea, Seoul, South Korea; Department of Internal Medicine, Seoul St. Mary’s Hospital, 505 Banpo-Dong, Seocho-Ku, 137-040 Seoul Korea

After publication of this article [1] in the journal Stem Cell Research and Therapy, it was brought to our attention that the acknowledgement section was not complete. The correct acknowledgement section should read:

This research was supported by the Bio & Medical Technology Development Program of the National Research Foundation (NRF) funded by the Korean government (MEST) (No. 2012M3A9C6049783) and by a grant of the Korean Health Technology R&D Project, Ministry for Health & Welfare, Republic of Korea.(HI14C3417). The authors declare that they have no competing interests.
